# Biomechanical effects of screws of different materials on vertebra-pediculoplasty: a finite element study

**DOI:** 10.3389/fbioe.2023.1225925

**Published:** 2023-06-29

**Authors:** Yan-Ni Wang, Ya-Nan Ren, Jun Han, Chao Chen, Xun Sun, Ming-Yuan Di, Yi-Ming Dou, Xin-Long Ma, Zheng Wang, Cheng-Fei Du, Qiang Yang

**Affiliations:** ^1^ Department of Spine Surgery, Tianjin Hospital, Tianjin University, Tianjin, China; ^2^ Tianjin Key Laboratory for Advanced Mechatronic System Design and Intelligent Control, School of Mechanical Engineering, Tianjin University of Technology, Tianjin, China; ^3^ National Demonstration Center for Experimental Mechanical and Electrical Engineering Education, Tianjin University of Technology, Tianjin, China; ^4^ Department of Orthopaedics, Chinese People’s Liberation Army General Hospital, Beijing, China

**Keywords:** screw, finite element analysis, biomechanics, PEEK, vertebra-pediculoplasty

## Abstract

**Background:** The effects of cannulated screws made of polyetheretherketone (PEEK) on the biomechanical properties of the vertebral body during vertebra-pediculoplasty remain unclear. This study aimed to investigate whether PEEK screws have the potential to replace titanium alloy screws.

**Methods:** The surgical model of two different materials of screws was constructed using the finite element method. The biomechanical effects of the two models on the vertebral body under different working conditions were compared.

**Results:** ① The peak von Mises stress of PEEK screws was significantly lower than that of titanium screws, with a reduction ranging from 52% to 80%. ② The von Mises stress values for the injured T12 spine were similar for both materials. Additionally, the segmental range of motion and intervertebral disc pressure showed no significant difference between the two materials.

**Conclusion:** PEEK screws demonstrated advantages over titanium screws and may serve as a viable alternative for screw materials in vertebra-pediculoplasty.

## 1 Introduction

Vertebra-Pediculoplasty has emerged as a novel treatment approach for managing split and delayed osteoporotic vertebral fractures that were at risk of cement dislocation (Noritaka et al, 2021). It addressed the issue of poor clinical outcomes associated with balloon kyphoplasty for cleft osteoporotic vertebral fractures (Takahashi et al, 2019). The method involved using cannulated screws inserted into the cement block, in combination with balloon kyphoplasty, to create a “pedicle” (Noritaka et al, 2021). Traditional screws were primarily made of titanium, which offered excellent mechanical properties and good biocompatibility. However, their elastic modulus significantly differed from that of bone tissue, thereby increasing the risk of implant-related complications such as screw loosening or fracture, degeneration of adjacent segments, and long-term complications like pseudarthrosis (Zhang and Rong, 2020).

To overcome these limitations, this study proposed the use of polyether-ether-ketone (PEEK) material as an alternative to titanium alloy screws. PEEK has been extensively studied as an orthopedic implant material since the 1990 s (Kurtz and Devine, 2007). It has been a semi-crystalline polymer that exhibited excellent mechanical properties, favorable biocompatibility, X-ray penetrability, and other desirable physical and chemical properties, making it a promising material for orthopedic implants (Panayotov et al, 2016). Furthermore, its elastic modulus closely resembles that of normal human bone tissue, reducing stress-shielding effects (Zhao et al, 2020; Mrówka et al, 2021). Hence, this study aimed to evaluate the potential benefits of using PEEK screws in vertebra-pediculoplasty to minimize the risks associated with titanium alloys.

As vertebra-pediculoplasty was a relatively new method, the biomechanical effects in clinical practice remained unclear. Finite element (FE) analysis served as a valuable tool for assessing the biomechanical parameters of vertebral columns (Marien et al, 2017), and several studies have been conducted to evaluate the biomechanical properties of titanium and PEEK retention bars and spacers during surgery (Li et al, 2023). However, studies on titanium and PEEK screws have been limited to *in vitro* experiments (Lindtner et al, 2018; Stavros et al, 2020). Therefore, this research employed finite element analysis to compare the biomechanical effects of PEEK and titanium screws in vertebra-pediculoplasty, utilizing a finite element model of the human T11-L1 segment. The findings of this study may provide valuable theoretical guidance for the clinical application of screw materials.

## 2 Materials and methods

### 2.1 Establishment of normal thoracolumbar and osteoporotic fracture models

The CT data of a healthy 30-year-old male were imported into Mimics software (Materialise Technologies, Leuven, Belgium) to initially create a geometric model of the thoracolumbar spine (T11-L1). The thoracolumbar spine model was then imported into 3-Matic (Materialise Technologies, Leuven, Belgium) for individual processing of each vertebral body, resulting in a more accurate model structure in Geomagic software (Geomagic Inc., North Carolina, United States). The model was further processed in HyperMesh software (Altair Engineering Corp, Michigan, United States) for meshing, material property assignment, and assembly. Finally, the model was imported into Abaqus software (Dassault Systemes, PA, United States) for calculations and analysis (Tan et al, 2021). The elastic modulus of the osteoporotic vertebral structures was determined based on POLIKEIT et al (POLIKEIT et al, 2003), and specific material properties were determined according to previous studies (SHIM et al, 2008), as shown in Table 1.

**TABLE 1:** Material properties of thoracolumbar spine and screws (POLIKEIT et al, 2003; Stavros et al, 2020; Tan et al, 2021).

Component	Young’s modulus (MPa) Osteoporosis (normal)	Poisson’s ratio Osteoporosis (normal)	Element type
Cortical	8,040 (12,000)	0.3 (0.3)	C3D8R
Cancellous	34 (100)	0.2 (0.3)	C3D4
Posterior element	2,345 (3,500)	0.25 (0.3)	C3D4
Endplate	670 (1,000)	0.4 (0.4)	C3D8R
bone cement	3,000	0.4	C3D4
Titanium screws	110,000	0.28	C3D4
PEEK screws	3,600	0.25	C3D4
Nucleus pulposus	Mooney-Rivlin, C1 = 0.18, C2 = 0.03		C3D8RH
Annulus fibers	Calibrated stress-strain curves		Spring
Facet cartilage	Neo-Hookean, C10 = 2		C3D8RH
Annulus ground	Mooney-Rivlin, C1 = 0.18, C2 = 0.045		C3D8RH
Ligament	Calibrated deflection–force curves		Spring

### 2.2 Establishment of surgical model

**FIGURE 1:**
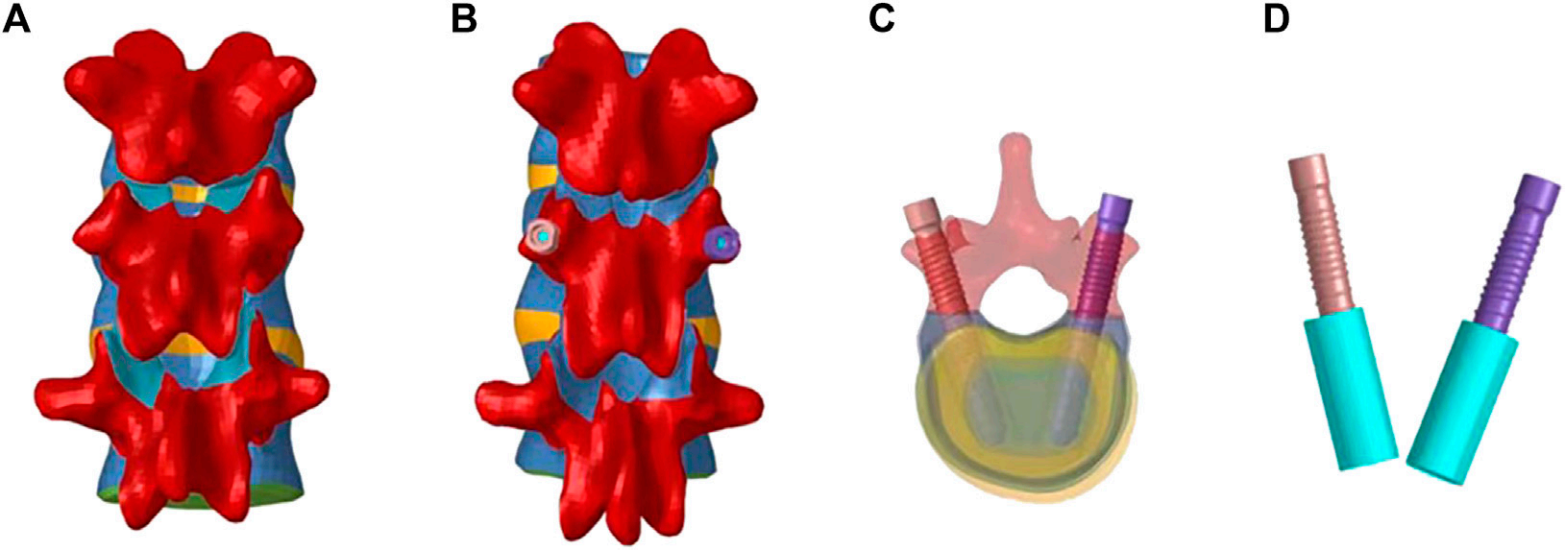
Establishment of the model after screw operation. **(A)**: Schematic diagram before T11-L1; **(B)**: Schematic diagram of T11-L1; **(C)**: Screw placement; **(D)**: Screw-cement model.

The hollow lateral screw geometry was created in SolidWorks and imported into HyperMesh to assemble it with the vertebral body. The vertebral body-screw connection was simulated using a “binding” constraint, completing the vertebral screw fusion model. Two postoperative models of different materials (titanium and PEEK) for screw placement into the vertebral body were established based on vertebra-pediculoplasty. The cannulated screws used were 6.5 mm in diameter and 50 mm long. During the operation, bone cement was injected into the vertebral body through the hollow screw, and it diffused around the side hole of the screw, wrapping the screw evenly in a cylindrical shape (WANG et al, 2014). Each cannulated screw was injected with 2 ml of bone cement, and a cylindrical block with a radius of 8 mm and a height of 9.95 mm was created in SolidWorks to simulate the bone cement block. The screws of the two different materials had the same structure and shape. The establishment of the surgical operation model is illustrated in Figure 1.

### 2.3 Loads and boundary conditions

A follower load of 500 N was applied to the upper surface of the T11 vertebral body to simulate physiological compressive loading. A moment load of 7.5 N m was applied to the T11 vertebral body to simulate forward flexion, back extension, lateral bending, and axial rotational motion. During loading, all degrees of freedom of the lower surface of the L1 vertebral body were constrained. (LIAO et al, 2017; DU et al, 2021).

### 2.4 Main outcome indicators

The maximum von Mises stresses of the screws and the injured T12 vertebral structure were compared for each model with different materials under various gestures such as flexion, extension, left bending, right bending, left rotation, and right rotation. Additionally, the segmental range of motion and intervertebral disc pressure were also evaluated.

## 3 Results

### 3.1 Verification of the normal thoracolumbar vertebrae finite element model

The range of motion (ROM) of the vertebral body was calculated under different postures. The ROMs of the T11-T12 segments were found to be 7.4°, 8.9°, and 4.6° for flexion and extension, lateral bending, and axial rotation, respectively. Similarly, the ROMs of the T12-L1 segments were 7.2°, 8.7°, and 3.8° for the corresponding postures. These results were compared with previous experimental data, and they were consistent with the findings reported in the literature (PANJABI et al, 1994; LIANG et al, 2015).

### 3.2 Maximum stress results of the screw

**FIGURE 2:**
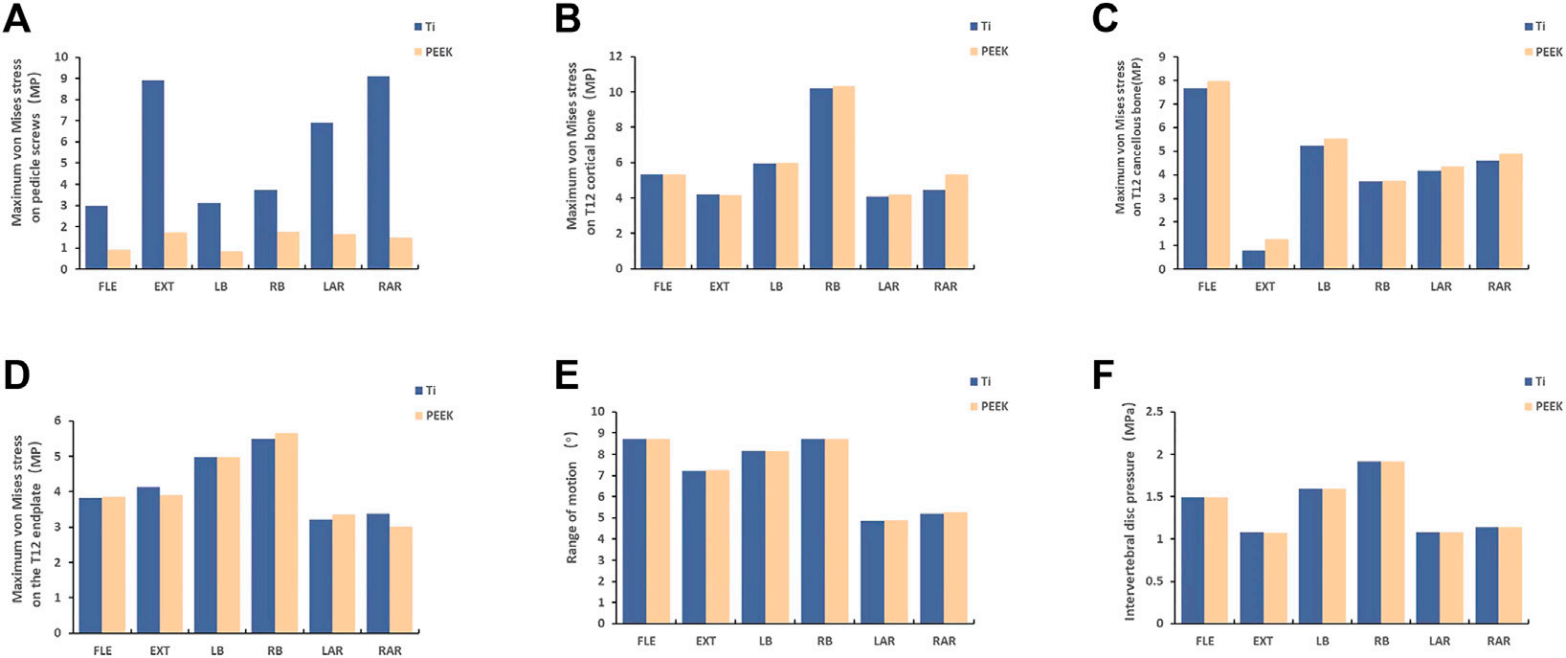
The stress results of the structure of the vertebra body [**(A)** The result of stress on pedicle screw; **(B, C, D)**: The stress results of the T12 structure of the injured vertebra; **(E, F)**: the result of the segmental range of motion and intervertebral disc pressure; FL = flexion, EX = extension, LB = left bending, RB = right bending, LAR = left axial rotation, and RAR = right axial rotation].

In the six models, the peak von Mises stress of the PEEK screws was 17.52 MPa, 9.125 MPa, 16.66 MPa, 8.48 MPa, 14.94 MPa, and 17.8 MPa. On the other hand, the peak stress of the titanium alloy screws was 89.03 MPa, 29.93 MPa, 69.06 MPa, 31.37 MPa, 90.88 MPa, and 37.48 MPa (e.g., Figure 2A). Upon comparison, it was observed that the maximum von Mises stress on PEEK screws was significantly lower than that on titanium screws.

### 3.3 Maximum Stress Analysis of Injured Vertebral T12 Structure and Analysis of Segmental Range of Motion and Intervertebral Disc Pressure

There were no significant differences in the maximum stress of the T12 cortical bone in the injured vertebra when using PEEK screws compared to titanium alloy screws under the six different states. Similarly, no noticeable differences were found in the maximum stress of the T12 cancellous bone or the analysis of the endplates (e.g. Figure 2B–D). Furthermore, the analysis of the range of motion of the vertebral body and the intervertebral disc pressure in the T11-L1 segment yielded similar results (e.g. Figure 2E, F). Upon comparing the results of the finite element analysis, it was concluded that different materials have minimal impact on the vertebral body.

## 4 Discussion

Due to its unique properties, PEEK has gained significant interest in bone implant research. The use of PEEK in orthopedic screws offers a promising avenue for exploration. In this study, we conducted a simulation-based analysis to assess the potential of PEEK as a substitute for the conventional titanium alloy used in screw fabrication. PEEK is a semi-crystalline polymer with excellent properties such as high modulus, melting point, processing performance, and strength (KULKARNI et al, 2007). Its elastic modulus closely resembles cortical bone, which reduces stress-shielding effects (MO et al, 2019). Additionally, PEEK is radiolucent, biocompatible, and does not cause artifacts during magnetic resonance scanning.

Recent efforts have focused on optimizing the mechanical and biological properties of PEEK through various methods such as 3D printing, coating, chemical modification, and the introduction of bioactive or antibacterial substances (Chen et al, 2022). These modifications aim to enhance the overall properties of PEEK and facilitate the treatment of bone injuries, making PEEK materials a promising option for lumbar spine repair.

In recent years, numerous studies have explored the factors that influence screw stability, including screw diameter, shape, length, thread shape, implantation method, angle, and combination (ABSHIRE et al, 2001; KINER et al, 2008; SENGUPTA and Herkowitz, 2012; Karami et al, 2015; JENDOUBI et al, 2018; NAKASHIMA et al, 2019). The current study investigates the effect of screw material in finite element analysis to provide further insights.

By comparing the von Mises stress of screws made from different materials under different vertebral body motions, our analysis reveals that PEEK screws have a significant advantage in reducing peak stress compared to titanium alloy screws. Specifically, the range of reduction observed in the von Mises stress with PEEK screws ranges between 52% and 80%. The observed reduction in peak stress indicates that the use of PEEK screws may lead to reduce the incidence of screw loosening, thereby establishing its potential as a promising alternative material. Some experiments have confirmed the idea that PEEK screws have a low risk of loosening. Richard Lintner et al (Lindtner et al, 2018) conducted cyclic loading tests on ten cadaveric lumbar vertebrae to compare the performance of carbon fiber-reinforced PEEK (CF/PEEK) and standard titanium pedicle screws in reducing screw loosening. The study found that PEEK and CF/PEEK screw/rod configurations had a significant advantage over titanium screws in reducing screw loosening. Similarly, Stavros Oikonomidis (Stavros et al, 2020) conducted cyclic loading tests on ten freshly frozen human cadaveric lumbar vertebrae to investigate the loosening rate of pedicle screws made of CFR/PEEK compared to titanium. The study concluded that the use of CFR/PEEK pedicle screws could reduce the rate of screw loosening. Further investigation is warranted to compensate for the lack of clinical studies using pedicle screws made of PEEK. One avenue for exploration is to compare relevant trials involving PEEK rods. Qian Jiaming et al (QIAN et al, 2022) and Huang Weimin et al (Huang et al, 2016) conducted follow-up studies for 6 months and 2 years, respectively, on patients who underwent posterior lumbar pedicle internal fixation and multi-level fixation using PEEK material. The results of the studies showed no instances of screw fracture or loosening during the respective follow-up periods. Although further clinical follow-up studies are required to ascertain the superiority of PEEK screws over other materials in preventing screw loosening, recent research suggests that PEEK screws may have similar benefits to PEEK rods in this regard.

**FIGURE 3:**
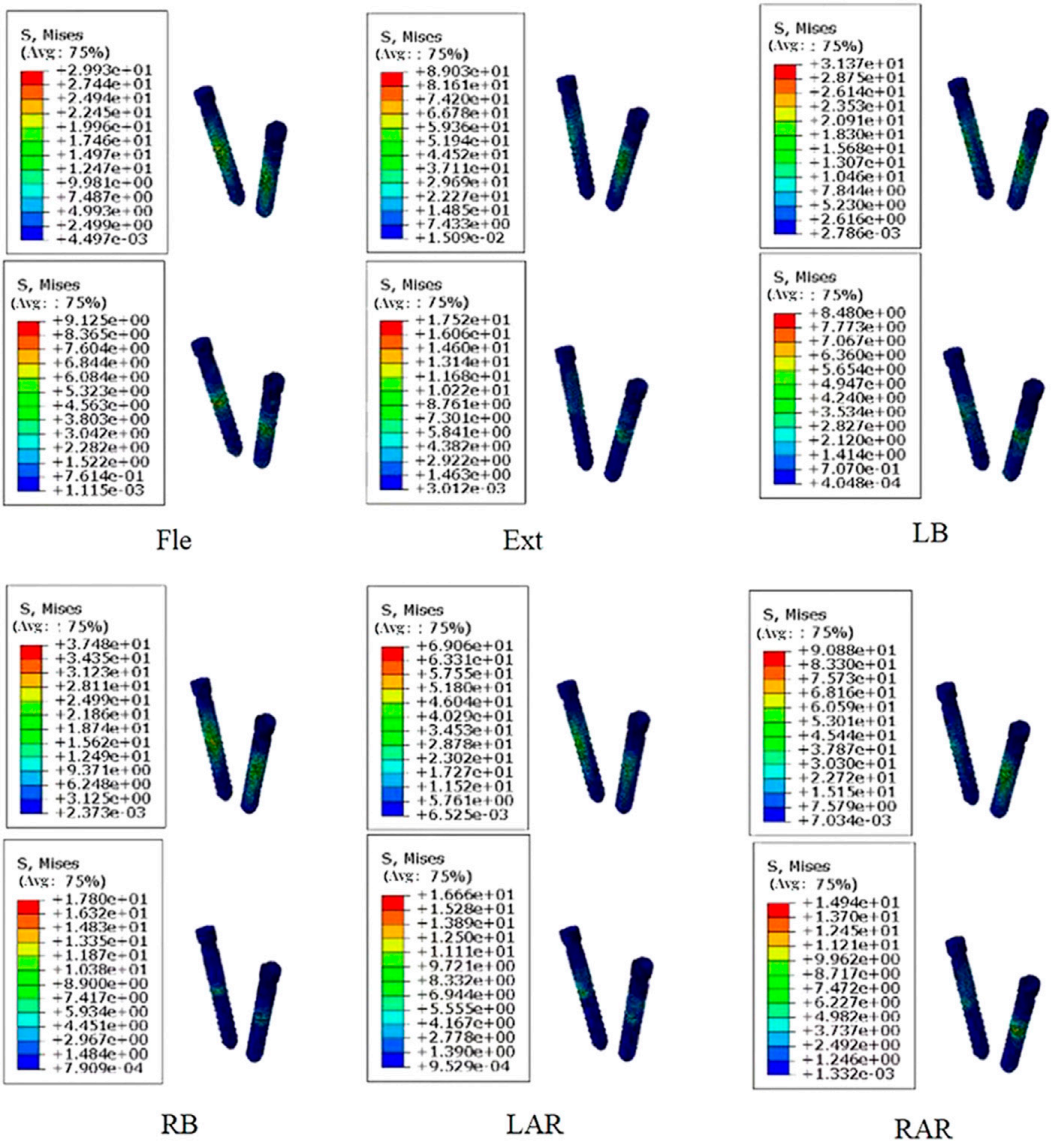
Comparison of stress distribution of screws made of two materials under different loading directions. (The figure shows titanium alloy material at the top and PEEK material at the bottom).

When assessing the risk of screw fracture, it is important to consider the ratio of peak stress to yield stress rather than focusing solely on stress magnitude. The ratio of peak stress to yield stress for PEEK screws ranged from 8% to 17%, while for titanium screws, it fell within the range of 3%–12% (PEEK: 100 MPa, titanium: 750 MPa). The higher percentage for PEEK screws suggests a potential escalation of breakage risk (e.g., Figure 3), consistent with prior findings by FAN et al (FAN et al, 2021). However, there have been no reported cases of PEEK rod fracture, possibly due to the load experienced under physiological conditions being insufficient to cause rupture. Consequently, screw breakage is unlikely to occur.

Previous investigations have examined the load transmission properties of titanium alloy posterior screw rod systems and PEEK screws in posterolateral lumbar fixation. These studies indicated that the titanium alloy system can transmit approximately 67% of the axial compressive load, while the natural upright state can bear only about 20% of the load (CUNNINGHAM and POLLY, 2002; AHN et al, 2008). PEEK screws have favorable characteristics such as biocompatibility, radiolucency, and a lower elastic modulus compared to titanium alloy screws. These characteristics allow PEEK screws to transfer more load to the front column, improving the load distribution between the front and rear columns. A finite element study by GARNET et al (GORNET et al, 2011) supported this finding, demonstrating that a titanium rod bore at least 6% more load than a PEEK rod. This evidence suggests that the principal advantage of PEEK screws lies in mitigating stress concentration on the screw.

Next, we analyze the finite element results of the vertebral structure. The von Mises stress of the vertebral body, as well as the segmental Range of Motion and Intervertebral Disc Pressure, were obtained through finite element analysis under various conditions. The results show that the stability of the vertebral body remains largely unaffected. Similar findings have been reported in related investigations. Nomidis et al (Stavros et al, 2020) conducted biomechanical experiments using cadaveric specimens and found no macroscopic changes in the vertebral structure. Additionally, YEAGER et al (YEAGER et al, 2015) compared PEEK and titanium rods using human lumbar spine specimens and concluded that both materials offered comparable stability under different loading modes. While PEEK may not match the strength and rigidity of titanium alloys, it possesses adequate strength and rigidity to maintain spinal stability and endure physiological biomechanical demands. The elastic modulus of PEEK closely matches that of bone tissue, allowing PEEK screws to conform to micro-movements and deformations of interconnected spinal bones. This feature reduces the likelihood of stress concentration and ensures a secure connection. Moreover, PEEK demonstrates exceptional biocompatibility, minimizing the risk of inflammatory responses or tissue rejection. PEEK screws can integrate stably with the surrounding bone tissue, exhibiting biostability comparable to that of titanium screws. The adaptive nature of PEEK material to bone morphology enables it to establish minute biological interconnections with bone tissue, enhancing the stability of PEEK screw integration with the spinal bone and reducing the risk of loosening. However, the development of more ideal internal fixation materials warrants further exploration through basic scientific research and clinical trials.

Several limitations should be considered when interpreting the results of this study. Firstly, the finite element model used is based on theoretical numerical simulations and may not fully capture the complexity of the human spine system, as it does not account for factors such as cyclic loads and the influence of muscles. Secondly, the thoracic and lumbar spine models used are limited to a single subject, and the number of models is small, which may limit the generalizability of the findings. Lastly, this study represents a preliminary exploration of finite element analysis. Further research and exploration are necessary to establish a solid foundation for the long-term development of PEEK material in lumbar spine repair.

## 5 Conclusion

In conclusion, PEEK screws demonstrate comparable efficacy to titanium alloy screws in providing segmental stability post-surgery. Additionally, PEEK screws facilitated the prevention of loosening, which was a great clinical advantage. Moreover, the radiolucent nature of PEEK screws facilitates postoperative imaging without interfering with radiation therapy. Thus, the PEEK or PEEK composite material may emerge as a viable alternative for screw materials in clinical practice. (Sato et al, 2018).

## Data Availability

The original contributions presented in the study are included in the article/Supplementary Material, further inquiries can be directed to the corresponding authors.
